# Real‐world data on home end‐of‐life care for older adults with cancer: A retrospective claims data analysis

**DOI:** 10.1002/agm2.12246

**Published:** 2023-03-25

**Authors:** Yukio Suzuki, Soshi Dohmae, Kohei Ohyama, Taiga Chiba, Sachiko Nakagami, Etsuko Miyagi, Jun Shuri

**Affiliations:** ^1^ Department of Obstetrics and Gynecology Yokohama City University Graduate School of Medicine Yokohama Japan; ^2^ Medical Policy Division, Medical Care Bureau, City of Yokohama Yokohama Japan; ^3^ Division of Gynecologic Oncology, Department of Obstetrics and Gynecology Columbia University Vagelos College of Physicians and Surgeons New York New York USA

**Keywords:** administrative database, cancer, home end‐of‐life care, home medical care, super‐aged society

## Abstract

**Background:**

Cancer incidence is expected to increase with population aging, making the availability of places for treating patients with terminal cancer a pressing issue. However, little is known about the actual state of home end‐of‐life care (HEC) in Japan.

**Objective:**

The objective of this study was to examine the real‐world state of HEC for older adults with cancer.

**Methods:**

The Yokohama Original Medical Database was used to identify the cohort. Data of target patients was extracted based on three criteria: age ≥65 years, malignant neoplasm diagnosis, and having a specific billing code of HEC. Multivariable linear and logistic regression models were used to evaluate the association between age groups and HEC services or outcome indexes.

**Results:**

Overall, 1323 people (554 and 769 aged < 80 and ≥ 80 years, respectively; men, 59.2%) had planned to receive HEC. The < 80 years group had more frequent emergent home visits than the ≥ 80‐year group (*P* < 0.001), but the number of monthly home visits was similar between the two groups (*P* = 0.267). The rate of emergent admission was 5.9% in the ≥ 80‐year group, which was higher than that in the < 80‐year group (3.1%; *P* = 0.018). Conversely, the rates of central venous nutrition and opioid use were higher in the < 80‐year group than those in the ≥ 80‐year group.

**Conclusions:**

This study reported patterns of use of HEC among older adults with cancer in the terminal stage. Our findings may provide the basis for providing HEC for older adults with cancer.

## Graphical abstract

There is a trend for an increase in the number of patients with cancer aged ≥ 65 years who want to die in their homes. Patients with terminal cancer aged ≥ 80 years were less dependent on home medical care than patients aged < 80 years.

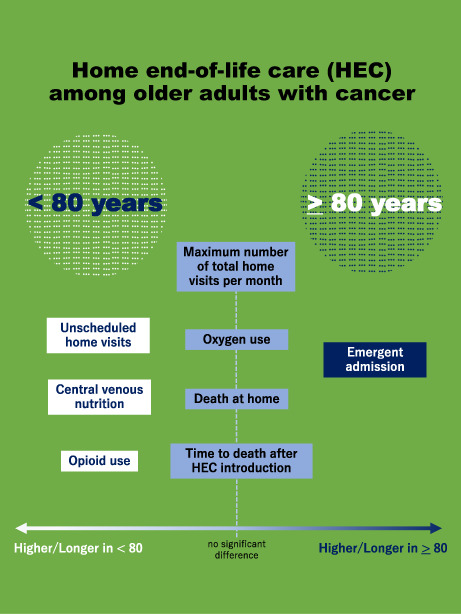

## 1 INTRODUCTION

Japan is unprecedently moving toward being a super‐aged society. By 2025, > 30% of Japanese people will be older than 65 years and approximately 18% will be older than 75 years.^1^ Moreover, by 2035, according to the estimation of the Cabinet Office in Japan, > 20% of Japanese people will be older than 75 years.^1^ The same situation is predicted to occur in Yokohama City, which is the most populous city in Japan after 23 districts in Tokyo and has a population of 3.74 million. Yokohama City (area, 435 km^2^) is approximately 30 minutes away from central Tokyo by train and represents a corner of the Tokyo Metropolitan area. Updated vital statistics predict that the percentage of older individuals in Yokohama City will exceed 25% in 2025 and 30% in 2035.^2^ The proportions of patients aged > 65 years who died from cancer (from 2014 to 2015) were 83.1% in Yokohama City^3^ and 84.2% nationally according to data from the National Cancer Center.^2^ With the growth in the aging population, the proportion of older adults expected to die from cancer is estimated to increase to 88.2% by 2025.^3^ Thus, it is important to focus on cancer care for older adults.

Most patients with cancer in Japan have so far spent the last moments of their lives in hospitals. However, due to the abovementioned factors, it is anticipated that there will be bed shortages for end‐of‐life care of patients with terminal stage cancer, and meeting this increased demand for older adults with cancer is a vital social concern. Therefore, a community‐based integrated care system is being promoted as an important health care initiative in Japan. However, little is known about the actual burden of home visits on a family doctor in charge of palliative care, which depends on the situation of the patient's need. Moreover, real‐world data on the overall survival after the transition to home medical care are limited. Although the government values home medical care and community‐based integrated care systems for meeting the urgent needs in Japan, pain control and other palliative treatments become challenging when patients with cancer are accepted into family clinics, and the doctors need to have extensive experience of palliative care. Thus, the number of doctors who accept patients with cancer for end‐of‐life care is limited. To motivate family doctors to accept such patients, details of the burden of home medical care for patients with cancer need to be provided. In contrast to the Japanese situation, in Canada, which is known as a well‐organized country in the implementation of palliative care, the location of death at home exceeds 60% in older adults.^4^ In addition, in many studies, the effectiveness of end‐of‐life care has been proven through population‐based studies.^4^, ^5^ The community‐based care is recommended to achieve better palliative care.^6^

Awareness of the current situation of home end‐of‐life care (HEC) is indispensable to understand future supply and demand balance. Thus, we aimed to evaluate the current situation of HEC for older adults with cancer in Yokohama City using an original administrative database to achieve further dissemination of HEC. Unlike existing medical databases, such as registry records of specific diseases, this database was developed for policymaking at the local government level. It is expected to be the first step of evidence‐based policymaking (EBPM) through the gathering of information on medical policy problems, which was recommended by The Center for Government Excellence at Johns Hopkins University.^7^

## 2 METHODS

### 2.1 Database and setting

Yokohama City is the most populous city in Japan and is governed by the local government for the Greater Tokyo Area, which includes the Tokyo metropolis. Yokohama City has a population of approximately 3.75 million (January 2021), and the population distribution is as follows: individuals aged < 15 yearfs, 11.9%; 15–64 years, 62.8%; and ≥ 65 years, 25.2% (January 2021).^2^ The Yokohama Original Medical Database (YoMDB)^8^ was built by Yokohama City after approval from the Information Disclosure and Personal Information Protection Review Board based on the Yokohama City Ordinance. This large medical invoice database can only be accessed by administrative officers from departments in charge of medical care and includes data on all residents of Yokohama City who have the following three government‐funded insurance types: National Health Insurance, Long Life Medical Care System, and Public Assistance. The National Health Insurance is a system for farmers, self‐employed people, and other such individuals. The Long Life Medical Care System is the system for all individuals aged ≥ 75 years. The personal numbers of insurance and public assistance are hashed to protect the identity of individuals, and information that could reveal an individual's identity, such as their name and treatment details, is deleted to make the database secure.

This database includes 68.3% of Yokohama City residents aged 65–69 years; 84.1% of residents aged 70–74 years, with 99.7% of them aged 75–79 years; and 98.6% of residents aged > 80 years. Hence, it has a strong representation of the older adults and is an especially reliable database for those older than 75 years.

### 2.2 Case selection

The data from 2014 and 2015 included those of 2,486,834 people and 29,411,895 medical invoices. Target patients were selected based on three criteria: age ≥ 65 years, death caused by a malignant neoplasm based on International Classification of Diseases (ICD)‐10 codes (C00‐C97), and additional HEC charges. The additional charge could have been applied by an insurance‐participating medical facility when planned/emergent home medical care and nursing visits were provided more than twice for a total of 15 days (within 14 days before the day of death).

### 2.3 Statistical analyses and definition of variables

Patients' demographics, including age, sex, insurance type, cancer type, and institution type, were collected from the database. In this study, we divided the patients into the < 80 years and ≥ 80 years groups according to their age. The age of 80 years is commonly used as one of the criteria when doctors make a decision for the options of cancer treatment in actual clinical practice.^9^ Cancer type was classified as follows: oral and oropharyngeal (ICD‐10 code C00‐C14 and C32), esophageal and gastric (C15 and C16), colon (C18‐C20), hepatobiliary and pancreatic (C22‐C25), lung (C33‐C34), skin (C43‐C44), breast (C50), gynecologic (C53‐C56), prostate (C61), urinary tract (C64‐C68), brain (C70‐C72), thyroid (C73), and blood (C81‐C85, C88‐C96) cancers. Institution types were classified into clinics and hospitals based on the definition in the Medical Care Act in Japan. Clinics have less than 20 inpatient beds, whereas hospitals have 20 or more beds.

HEC was classified based on the three types of home visits as follows: planned home visits (p‐HVs), a home visit with a request from a patient (urgent home visits [u‐HVs]), and emergent home visits (e‐HVs). The difference between u‐HV and e‐HV is if a family doctor visits the patient's home right after a request call from a patient. Most of the u‐HVs are the cases of minor issues or mild symptoms, on the contrary, most of e‐HVs are the cases of major issues or moderate to severe symptoms which needs the doctor to visit immediately. Furthermore, e‐HVs were divided according to the timing of the visit as follows: daytime e‐HV (visit during clinic hours), midnight e‐HV (visit from 10:00 pm to 06:00 am), and night/holiday e‐HV (visit at a time other than clinic hours and midnight). These classifications were consistent with the medical fee point system in Japan.

Medical fee point data related to home medical care (coded as C000 to C171‐2 in the Japanese original list of Medical Fee Points) were obtained based on the invoice of medical treatment for the target patients during the 2‐year period and were analyzed. The three types of home visits were coded as follows: p‐HVs was coded as C001, u‐HVs as C000, and e‐HVs as C0001‐C0005. Admission was defined as the claim of the basic admission fee (coded as A100‐109 in the Japanese original list of Medical Fee Points) or special admission fee (coded as A300‐317 in the Japanese original list of Medical Fee Points), including admission to the critical care unit, intensive care unit, high care unit, palliative care unit, and community‐based integrated care unit.

Emergent admission could not be directly confirmed due to the payment system. Cases with data on planned/emergent home medical care fee and hospitalization fee in the following month were referred to as cases of emergent hospitalization. Survival time at home was defined as the period from the first home medical care event to the month in which the additional fee for end‐of‐life care was charged.

Comparisons were made between the groups of patients aged < 80 years and ≥ 80 years. Multivariable linear and logistic regression model were used to evaluate associations between age groups and HEC services or outcome indexes. Age group, sex, and location of institution were included in these models. For the survival analysis, a log‐rank test stratified by age group was used. In addition, we used a Cox proportional hazards model to estimate a hazard ratio (HR) with 95% confidence interval (CI) and evaluate factors associated with time to death after HEC introduction. Age group, sex, and location of institution were included in the Cox hazard model. Insurance type was not included in the model because this type was strongly connected to the recipient's age. Chi‐square test was used to compare baseline characteristics between age groups. The *P* values < 0.05 were considered statistically significant. All statistical analyses were performed using Statistical Package for the Social Sciences, version 28 (International Business Machines Corporation, Armonk, NY, USA).

## 3 RESULTS

### 3.1 Baseline characteristics

The complete data for 2014 and 2015 included those of 1,239,426 and 1,247,408 people and 14,467,489 and 14,944,496 medical invoices, respectively. Our algorithm showed that 1323 people had planned to receive HEC. The baseline characteristics of the patients are shown in Table 1.

**TABLE 1:** Patient baseline characteristics.

	All	< 80 y	≥ 80 y	*P* value
	n (%)	n (%)	n (%)	
Number	1323 (100)	554 (41.9)	769 (58.1)	
Sex				0.001
Male	783 (59.2)	358 (64.6)	425 (55.3)	
Female	540 (40.8)	196 (35.4)	344 (44.7)	
Insurance types				< 0.001
National Health Insurance	306 (23.1)	306 (55.2)	0 (0.0)	
Long Life Medical Care System	966 (73.0)	218 (39.4)	748 (97.3)	
Public Assistance	51 (3.9)	30 (5.4)	21 (3.7)	
Cancer types				0.383
Lung	221 (16.7)	99 (17.9)	122 (15.9)	
Gastric	208 (15.7)	85 (15.3)	123 (16.0)	
Colon	177 (13.4)	72 (13.0)	105 (13.7)	
Pancreatic	103 (7.8)	47 (8.5)	56 (7.3)	
Liver	85 (6.4)	30 (5.4)	55 (7.2)	
Prostate	71 (5.4)	30 (5.4)	41 (5.3)	
Urinary tract	62 (4.7)	22 (4.0)	40 (5.2)	
Biliary tract	60 (4.5)	19 (3.4)	41 (5.3)	
Blood	44 (3.3)	14 (2.5)	30 (3.9)	
Esophageal	43 (3.3)	21 (3.8)	22 (2.9)	
Gynecologic	35 (2.6)	19 (3.4)	16 (2.1)	
Breast	34 (2.6)	17 (3.1)	17 (2.2)	
Others (including metastatic cancer)	180 (13.6)	79 (14.3)	101 (13.1)	
Institution				0.890
Clinic	1250 (94.5)	524 (94.6)	726 (94.4)	
Hospital	73 (5.5)	30 (5.4)	43 (5.6)	
Location of institution				< 0.001
In Yokohama City	1200 (90.7)	518 (93.5)	682 (88.7)	
Outside of Yokohama City	123 (9.3)	36 (6.5)	87 (11.3)	

The proportions of male and female patients were 59.2% and 40.8%, respectively. The numbers of medical invoices for planned and unscheduled home visits (per 1000 people) in 2014 and 2015 obtained from the YoMDB were as follows: 554 for residents aged < 80 years and 769 for residents aged ≥ 80 years. The proportion of men was higher in the < 80 years group than in the ≥ 80 years group (*P* = 0.001). Insurance types were as follows: 306 (23.1%), 966 (73.0%), and 51 (3.9%) patients received National Health Insurance, Medical Care System for Older Senior Treatment, and Public Assistance, respectively. The types of cancers were as follows: 221 (16.7%), 208 (15.7%), 177 (13.4%), 103 (7.8%), 85 (6.4%), and 71 (5.4%) patients had lung, gastric, colon, pancreatic, liver, and prostate cancer, respectively (Table 1). Clinics were largely responsible for patients receiving HEC (94.5%).

### 3.2 Results of home end‐of‐life care

The clinical services and outcome index of HEC are shown in Table 2. The average number of composite unscheduled HVs was 1.9 times per person‐month.

**TABLE 2:** Clinical services and outcome index regarding HEC with multivariable regression models.

The clinical services and outcome index regarding HEC	All	< 80 y	≥ 80 y	*β* (95% CI)	*P* value
	(n = 1323)	(n = 554)	(n = 769)		
Maximum number of p‐HVs, u‐HVs, and e‐HVs (times/mo, average ± SD)	5.1 ± 1.6	4.9 ± 2.5	5.1 ± 2.6	0.208 (−0.076, 0.492)	0.151
Composite unscheduled HVs (times/person‐month, average ± SD)	1.9 ± 1.3	2.0 ± 1.4	1.8 ± 1.3	−0.185 (−0.331, −0.039)	0.013
u‐HVs (times/person‐month, average ± SD)	1.3 ± 1.1	1.5 ± 1.1	1.2 ± 1.1	−0.055 (−0.176, 0.066)	0.377
Overall e‐HVs (times/person‐month, average + SD)	0.5 ± 0.7	0.6 ± 0.7	0.5 ± 0.6	−0.130 (−0.206, −0.055)	0.001
Daytime e‐HVs (times/person‐month, average + SD)	0.1 ± 0.4	0.2 ± 0.4	0.1 ± 0.3	−0.039 (−0.077, 0.000)	0.048
Night/holiday e‐HVs (times/person‐month, average + SD)	0.2 ± 0.4	0.2 ± 0.5	0.2 ± 0.4	−0.038 (−0.084, 0.008)	0.101
Midnight e‐HVs (times/person‐month, average + SD)	0.2 ± 0.4	0.2 ± 0.4	0.2 ± 0.4	−0.053 (−0.094, −0.012)	0.011

The clinical services and outcome index regarding HEC	All	< 80 y	≥ 80 y	aOR (95% CI)	*P* value
	(n = 1323)	(n = 554)	(n = 769)		
Emergent admission, n (%)	62 (4.7)	17 (3.1)	45 (5.9)	1.933 (1.089, 3.429)	0.024
Central venous nutrition, n (%)	90 (6.8)	48 (8.7)	42 (5.5)	0.586 (0.379, 0.907)	0.016
Oxygen use at home, n (%)	500 (37.8)	218 (39.4)	282 (36.7)	0.906 (0.722, 1.136)	0.390
Opioid use, n (%)	1014 (76.6)	476 (85.9)	538 (70.0)	0.402 (0.302, 0.536)	< 0.001
Four or more p‐HVs by a doctor or nurse in a week, n (%)	132 (10.0)	56 (10.1)	76 (9.9)	0.926 (0.640, 1.340)	0.684
Death at home, n (%)	1261 (95.3)	532 (96.0)	729 (94.8)	0.721 (0.423, 1.229)	0.721

*Note*: Daytime is defined as clinic hours. Midnight is defined as the time from 10:00 pm to 06:00 am. Night/holiday is defined as the time other than clinic hours and midnight. Composite unscheduled HVs is defined as a total number of u‐HVs and overall e‐HVs. *β* was a coefficient of age group calculated by a multiple linear regression model, which includes age group, sex, and location of institution. The aORs were adjusted by sex and location of institution.

Abbreviations: aOR, adjusted odds ratio; CI, confidence interval; e‐HVs, emergent home visits; HEC, home end‐of‐life care; p‐HVs, planned home visits; u‐HVs, urgent home visits.

The < 80 years group received more unscheduled HVs than the ≥ 80 years group (1.5 *vs*. 1.2 times/person‐month, *P* < 0.001), especially outside the outpatient clinic (*P* = 0.001). The rate of emergent admission was 5.9% in the ≥ 80 years group, which was higher than that in the < 80 years group (3.1%; *P* = 0.018). Conversely, the rates of central venous nutrition and opioid use were higher in the < 80 years group than those in the ≥ 80 years group. The need for more than 3 days of HVs and/or nursing visits per week, the rate of death in the patient's home, and oxygen use were not significantly different between the two groups.

**FIGURE 1:**
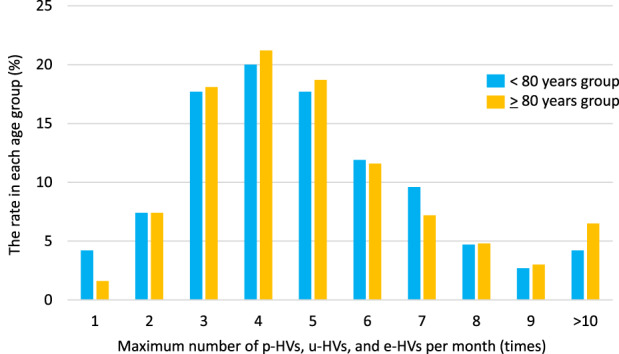
Maximum number of p‐HVs, u‐HVs, and e‐HVs per month within 6 months of death. The average number of home visits among all patients was 5.1 ± 2.6. There was no difference in the number of home visits per month between the < 80 years and ≥80 years groups (5.1 *vs*. 5.0, *P* = 0.267). p‐HVs, planned home visits; u‐HVs, urgent home visits; e‐HVs, emergent home visits.

The maximum numbers of p‐HVs, u‐HVs, and e‐HVs per month within 6 months from death are shown in Figure 1. The average number of home visits for all patients was 5.1 ± 2.6. There was no difference in the maximum number of home visits per month between the two groups (< 80 years group 4.9 months *vs.* ≥ 80 years group 5.1 months, *P* = 0.150).

### 3.3 Survival time after HEC introduction

**FIGURE 2:**
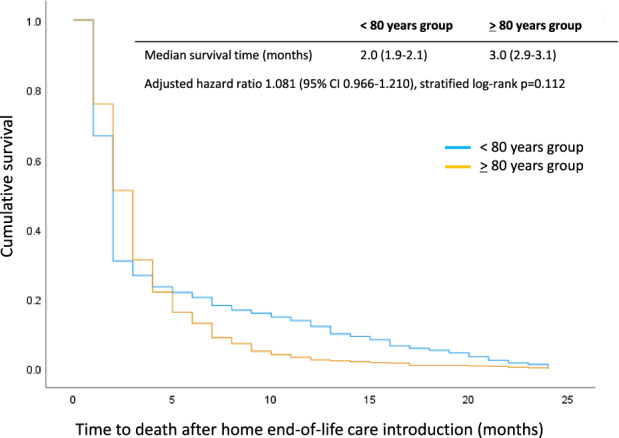
Kaplan–Meier estimated curves of the survival time after the introduction of home end‐of‐life care (HEC). CI, confidence interval.

The time to death after HEC introduction is shown in Figure 2. The median overall survival at home was 2.0 months (95% CI: 1.893–2.107) in <80 years group, while 3.0 months (95% CI: 2.854–3.146) in ≥ 80 years group (HR= 1.081, 95% CI: 0.966–1.210, *P* = 0.175). Cox proportional hazard model showed that being a women (HR= 0.859, 95% CI: 0.769–0.960, *P* = 0.007) was a significant factor to make survival time longer in the HEC setting.

## 4 DISCUSSION

To promote the concept of EBPM, we analyzed an original and large local government‐based medical database consisting of medical invoice data. We found that patients received HEC regardless of their cancer type. Furthermore, through our analysis, the necessary home medical care resources and monthly frequency of HVs were identified. This comprehensive database analysis with a high coverage of individuals aged ≥ 65 years showed that patients with terminal cancer older than 80 years were less dependent on home medical care than the patients aged younger than 80 years. To the best of our knowledge, this is the first such analysis performed using a large, local government‐based medical administrative database in Japan.

One of the significant contributions of this study was that it provided high‐value real‐world data on survival time after HEC introduction, which is difficult to analyze through medical invoice data in the Japanese system wherein survival time cannot be directly obtained. The YoMDB is strongly representative of the population of older adults because the insurance system in Japan focuses on this population.^8^ Moreover, the YoMDB is a highly comprehensive database for those older than 65 years. This universality is useful for EBPM. We targeted patients aged ≥ 65 years in this study because the proportion of patients aged ≥ 65 years who died of cancer was > 83%.^3^ Therefore, such a focused analysis was important from the viewpoint of advancing medical care in the near future for an aging society.

In the Japan HOspice and Palliative care Evaluation study, which was the first large nationwide study focusing on end‐of‐life care that surveyed bereaved families, home hospice care was found to be superior to end‐of‐life care at a cancer center in terms of the overall care satisfaction of the bereaved family, care evaluation scale scores,^10^ and good death inventory scores.^11^ In another cohort study, patients with cancer receiving HEC were found to have a better prognosis than those receiving hospital care.^12^

Older adults with advanced disease, especially patients with cancer, preferred HEC (odds ratio, 3.72).^13^ There is a trend for an increase in the number of patients with cancer aged ≥ 65 years who want to die in their homes.^13^ This finding is similar to that obtained in a previous Japanese study, which demonstrated that 72.8% of Japanese individuals would like to spend the end of their lives at home if they are diagnosed with cancer^14^; approximately 80% of those in their 70s desired to spend their last moments at home.^14^ However, 41.6% of respondents thought that this would be difficult to accomplish,^14^ which highlights the problems of social support and medical resource shortage.

In our study, more than 50% of older adults with cancer could spend 2 months or more at home. A retrospective study of home palliative care in 450 patients with advanced cancer in Japan showed poorer prognosis than that reported in our study.^15^ We believe that this difference arises from the differences in the nature of the hospital‐based medical record database. On the contrary, our administrative database had high coverage because it contained real‐world data. In addition, there might be patients out of this cohort who needed to be treated in either hospital or hospice at the terminal phase due to the severity and lack of care support by family. We have to interpret carefully the survival data because there could be selection bias when a patient, the family, and health care providers decide the place where the best place is for the patient.

The YoMDB is a useful administrative database for analyzing real‐world data as it contains data of local residents in Yokohama City. Health care policymaking is essential for ensuring a healthy society; thus, evidence‐based policies, similar to those used to treat a patient in a hospital or clinic, should be developed. There is an urgent need to develop human resources responsible for home medical care and a medical care provision system that can meet the increasing demand for HEC for older adults with cancer in the near future. A local government and local medical association that manage a community‐based integrated care system should proceed to increase the number of home care clinics involving cancer terminal care. An effective question to clinics might be as follows: “Why don't you start HEC from ≥ 80 years patients with cancer who have relatively mild symptoms?”

This study has several limitations. First, given the nature of the receipt database (it is based on a fixed code for the practice performed), we could not confirm the practice or outcome that we would like to have directly obtained. This may have resulted in some information bias. Second, because the reason for emergency hospitalization could not be correctly identified, it was difficult to analyze in detail whether palliation was acute, whether it was for respite purposes, or whether there was a different underlying requirement. Third, the analyses of medical invoice databases are limited because Japanese receipt data are originally used for the calculation of medical treatment fee, and analysis of the patient's condition and severity of underlying disease or comorbidity using these data was not possible. Last, it could solve selection bias if we could analyze a combined dataset which includes all the terminal stage patients treated at the hospital, hospice, and home.

## 5 CONCLUSIONS

This study reported patterns of use of HEC among older adults with cancer in the terminal stage. In areas where the population will continue to age further, such as Japan, the need for HEC for patients with cancer will definitely increase, and the development of social resources to meet this demand is an urgent social issue. Our results are valuable as they can be used as the basis for providing HEC through a community‐based integrated care system and will be useful for raising awareness and for human resource development. In particular, for medical institutions that intend to start HEC for patients with cancer, starting from older adults who have relatively mild symptoms could be a useful and concrete first step as these patients have a relatively low degree of medical dependence.

## AUTHOR CONTRIBUTIONS

All authors have made substantial contributions to the study and manuscript preparation. *Study design*: Suzuki. *Data collection*: Suzuki and Dohmae. *Data analysis*: Suzuki and Dohmae. *Data interpretation*: Suzuki, Ohyama, Miyagi, and Shuri. All authors have read and approved the final manuscript.

## FUNDING INFORMATION

Not applicable.

## CONFLICT OF INTEREST STATEMENT

The authors declare that they have no competing interests.

## ETHICS STATEMENT

This study protocol was approved by the Institutional Ethics Committee of Yokohama City University School of Medicine (B180700010). This study used an opt‐out system at the official website of Yokohama City instead of obtaining informed consent. The Information Disclosure and Personal Information Protection Review Board based on the Yokohama City Ordinance waived the informed consent. This study was conducted in compliance with the provisions of the Declaration of Helsinki (as revised in Brazil 2013).

## Data Availability

The datasets generated and analyzed during the current study are available only for officers in Medical Policy Division, Medical Care Bureau. No one outside of the section cannot use this database and generated dataset by the Yokohama City Ordinance. If someone would like to use this database, we can consider implementing collaborative research. The corresponding author is going to be contacted by someone who plans to use this database.
